# Automatic Facial Recognition of Williams-Beuren Syndrome Based on Deep Convolutional Neural Networks

**DOI:** 10.3389/fped.2021.648255

**Published:** 2021-05-19

**Authors:** Hui Liu, Zi-Hua Mo, Hang Yang, Zheng-Fu Zhang, Dian Hong, Long Wen, Min-Yin Lin, Ying-Yi Zheng, Zhi-Wei Zhang, Xiao-Wei Xu, Jian Zhuang, Shu-Shui Wang

**Affiliations:** ^1^Department of Pediatric Cardiology, Guangdong Provincial People's Hospital, Guangdong Academy of Medical Sciences, Guangdong Cardiovascular Institute, Guangzhou, China; ^2^Department of Radiology, Guangdong Provincial People's Hospital, Guangdong Academy of Medical Sciences, Guangzhou, China; ^3^Department of Radiology, Qingdao Central Hospital, Qingdao, China; ^4^Cardiac Center, Guangdong Women and Children Hospital, Guangzhou, China; ^5^Guangdong Provincial Key Laboratory of South China Structural Heart Disease, Guangdong Provincial People's Hospital, Guangdong Academy of Medical Sciences, Guangzhou, China; ^6^Department of Cardiac Surgery, Guangdong Provincial People's Hospital, Guangdong Academy of Medical Sciences, Guangdong Cardiovascular Institute, Guangzhou, China

**Keywords:** Williams-Beuren syndrome, genetic syndrome, convolutional neural networks, artificial intelligence, automated facial recognition

## Abstract

**Background:** Williams-Beuren syndrome (WBS) is a rare genetic syndrome with a characteristic “elfin” facial gestalt. The “elfin” facial characteristics include a broad forehead, periorbital puffiness, flat nasal bridge, short upturned nose, wide mouth, thick lips, and pointed chin. Recently, deep convolutional neural networks (CNNs) have been successfully applied to facial recognition for diagnosing genetic syndromes. However, there is little research on WBS facial recognition using deep CNNs.

**Objective:** The purpose of this study was to construct an automatic facial recognition model for WBS diagnosis based on deep CNNs.

**Methods:** The study enrolled 104 WBS children, 91 cases with other genetic syndromes, and 145 healthy children. The photo dataset used only one frontal facial photo from each participant. Five face recognition frameworks for WBS were constructed by adopting the VGG-16, VGG-19, ResNet-18, ResNet-34, and MobileNet-V2 architectures, respectively. ImageNet transfer learning was used to avoid over-fitting. The classification performance of the facial recognition models was assessed by five-fold cross validation, and comparison with human experts was performed.

**Results:** The five face recognition frameworks for WBS were constructed. The VGG-19 model achieved the best performance. The accuracy, precision, recall, F1 score, and area under curve (AUC) of the VGG-19 model were 92.7 ± 1.3%, 94.0 ± 5.6%, 81.7 ± 3.6%, 87.2 ± 2.0%, and 89.6 ± 1.3%, respectively. The highest accuracy, precision, recall, F1 score, and AUC of human experts were 82.1, 65.9, 85.6, 74.5, and 83.0%, respectively. The AUCs of each human expert were inferior to the AUCs of the VGG-16 (88.6 ± 3.5%), VGG-19 (89.6 ± 1.3%), ResNet-18 (83.6 ± 8.2%), and ResNet-34 (86.3 ± 4.9%) models.

**Conclusions:** This study highlighted the possibility of using deep CNNs for diagnosing WBS in clinical practice. The facial recognition framework based on VGG-19 could play a prominent role in WBS diagnosis. Transfer learning technology can help to construct facial recognition models of genetic syndromes with small-scale datasets.

## Introduction

Williams-Beuren Syndrome (WBS) is a rare genetic syndrome, with an occurrence of ~1 in 10,000 persons (1). The syndrome consists of a characteristic facial gestalt, cardiovascular abnormalities, intellectual disability, and hypercalcemia. WBS is caused by the deletion of ~1.5 million to 1.8 million base pairs on chromosome 7q11.23, which encompasses 26–28 genes (2). This multisystem disease can be confirmed by genetic testing, such as array comparative genomic hybridization, fluorescence *in situ* hybridization, quantitative real-time polymerase chain reaction, multiplex ligation-dependent probe amplification, and gene sequencing. Nowadays, genetic testing is readily available at major hospitals and cytogenetics laboratories, but it is not still a routine test. The diagnosis of WBS often begins when astute clinicians recognize the specific facial dysmorphism. The characteristic facial gestalt of WBS is described as “cute” or “elfin” (3). Facial characteristics include broad forehead, periorbital puffiness, flat nasal bridge, short upturned nose, long philtrum, wide mouth, thick lips, and pointed chin (4, 5). The facial gestalt is an important clue for recognizing WBS in clinical practice. Thus, it will be beneficial to exploit a precise computer-aided recognition tool for WBS diagnosis. Such an automated model could improve the initial diagnosis workflow, and provide valuable information to pediatricians.

Recently, convolutional neural networks (CNNs), as one aspect of artificial intelligence and deep learning, have become the dominant machine learning approach for computer vision, especially in facial recognition applications (6, 7). In 2014, Taigman et al. (8) presented a facial recognition system, DeepFace, and showed that deep CNNs could achieve human-level performance on the task of facial verification. Based on the automatic detection of facial features, CNNs have been incorporated into the assisted diagnosis of genetic syndromes. Several studies showed that CNN-based facial recognition models for genetic syndrome diagnosis achieved high accuracy (9–12). In 2019, Gurovich et al. (10) reported a deep CNN framework, DeepGestalt, trained on a dataset of over 17,000 pictures of faces representing more than 200 syndromes. The model achieved the top-10 accuracy of 91% for identifying the correct genetic syndrome. Although the DeepGestalt model lists WBS as a possible target syndrome, this model does not focus on WBS diagnosis.

In this study, five face recognition frameworks for WBS were constructed by adopting the VGG-16 (13), VGG-19 (13), ResNet-18 (14), ResNet-34 (14), and MobileNet-V2 (15) state-of-the-art CNN architectures. By comparing the performance of these five frameworks, the best one was obtained. The five WBS facial recognition frameworks were also evaluated by comparing with four human experts.

## Patients and Methods

### Patients and Facial Photos

**Figure 1:**
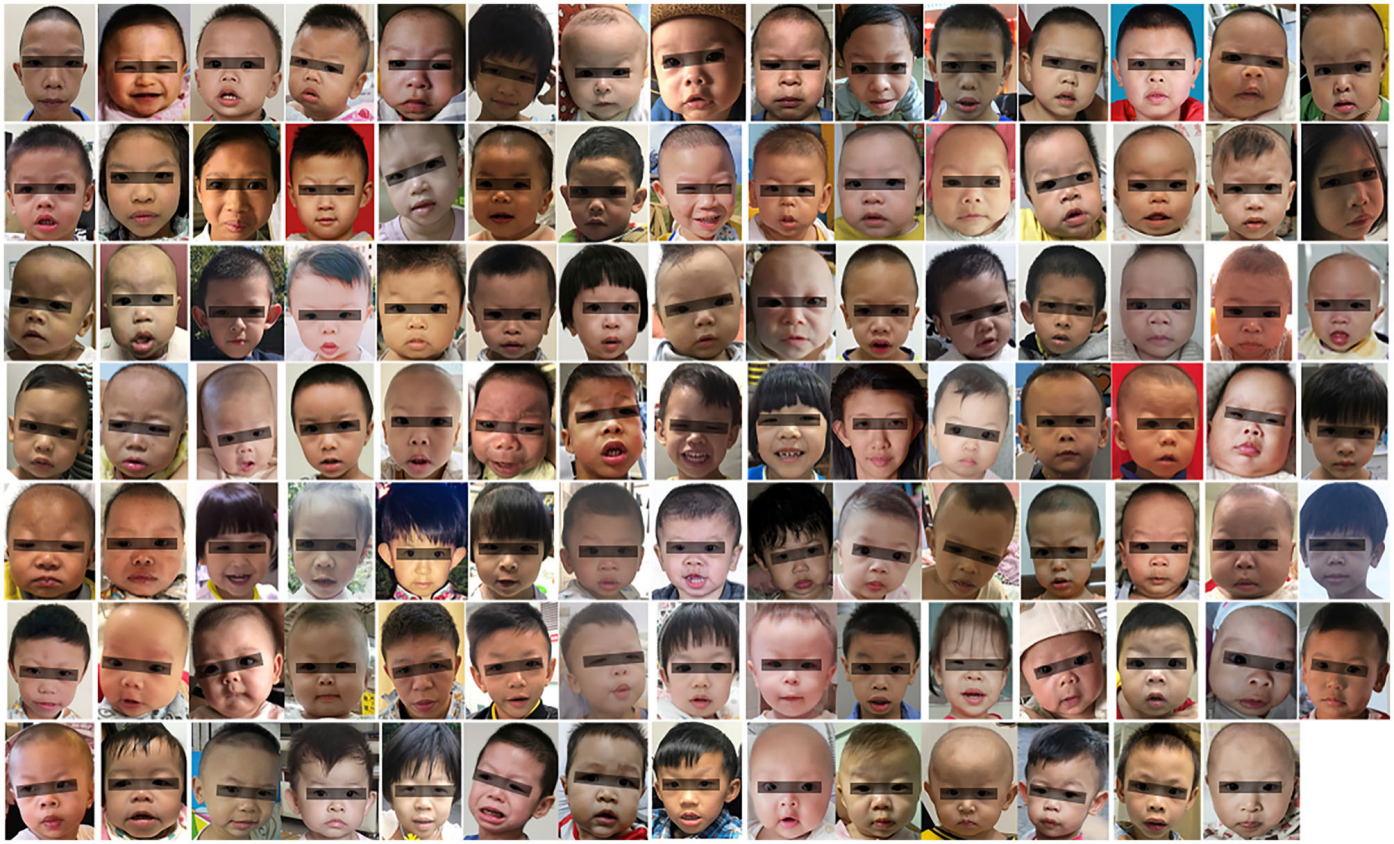
Facial appearance of WBS patients (104 cases). The black bar is used to protect privacy.

Images of 104 WBS patients and 236 control individuals were collected from the Guangdong Provincial People's Hospital from September 2017 to November 2020. The demographic characteristics of each participant are shown in Table 1. The control individuals included age-matched healthy children (*n* = 145), Noonan syndrome (*n* = 43), Down syndrome (*n* = 10), Loeys–Dietz syndrome (*n* = 4), DiGeorge syndrome (*n* = 3), Marfan's syndrome (*n* = 3), Alagille syndrome (*n* = 3), and other rare syndromes (*n* = 25). All facial photos were taken using digital cameras or smartphones with a resolution of more than 3,024 × 4,032 pixels. Three to ten frontal facial photos were collected for each participant. The photo dataset used only one frontal facial photo from each participant (Figure 1). WBS cases and patients with other genetic syndromes were confirmed by karyotype, fluorescence *in situ* hybridization, or next-generation sequencing. The healthy children were consulted by two child genetic disease experts to exclude the presence of any genetic syndrome. This study was approved by the Research Ethics Committee of Guangdong Provincial People's Hospital, Guangdong Academy of Medical Sciences (Project No. KY2020-033-01).

**Table 1:** Demographic characteristics of WBS patients and control individuals.

**Characteristic**	**WBS**	**Controls**	***P-*value**
Number of subjects	104	236	
Age at photograph (months)	32.98 ± 32.62	40.65 ± 43.45	0.074
Sex (male/female)	61/43	123/113	0.270

*Age is presented as the mean ± SD*.

### Pre-processing of images

The facial photos shown to human experts were malnually cropped to ensure that the face was in the middle and occupied 30–60% of the area of each image. These cropped photos were also inputted to the face recognition system for further recognition study. Automatic face detection and alignment were performed with the Multi-Task Convolutional Neural Network (MTCNN) (16). The MTCNN generated a facial image (224 × 224 pixels) with five facial landmarks (left eye, right eye, nose, left mouth corner, and right mouth corner). Then, the training dataset was augmented twice by rotation transformation, random cropping, horizontal flipping, and AutoAugment (17). Each pixel value of image was subtracted by the mean value and divided by the standard deviation for standardization. The pixel value of the image was scaled and normalized from 0 to 1.

### CNN Models Construction

Five deep CNN architectures (VGG-16, VGG-19, ResNet-18, ResNet-34, and MobileNet-V2) were used to build five WBS face recognition frameworks (Table 2). VGGNet (13) is a deep CNN architecture developed by the Visual Geometry Group (VGG) at the University of Oxford. VGG-16 consists of 13 convolution layers and three fully connected layers, and VGG-19 includes 16 convolution layers and three fully connected layers. ResNet (14) is a residual network composed of residual blocks, where each block is a stack of convolutional layers. In addition to the direct connection of convolution layers, ResNet has a shortcut connection path between the input of a residual block and its own output. ResNet-18 includes 16 convolution layers and ResNet-34 includes 32 convolution layers. MobileNet-V2 (15) uses an inverted residual structure, where the shortcut connections are between the thin bottleneck layers. The number of parameters in each CNN architecture is shown in Table 2. The number of parameters refers to the total number of weights of the network, which determine the spatial complexity of the network.

**Table 2:** Number of parameters for the five deep CNN architectures.

**CNN architectures**	**Number of parameters (in millions)**
VGG-16	138
VGG-19	144
ResNet-18	12
ResNet-34	22
MobileNet-V2	4

In the experiment, five-fold cross-validation was adopted. The photo data were randomly split into five subsets (Table 3), and the three classes (WBS, other syndromes, and healthy children) were equally distributed in each subset. In each fold, the initialization weights of each CNN model were obtained through training on ImageNet (18) using transfer learning. The last layer was replaced with a fully connected layer with two outputs for binary classification. Then, the models were fine-tuned on the photo dataset. The batch-size was 16, and the learning rate was 0.01. The training ran for 100 epochs. The stochastic gradient descent optimization algorithm (19) was used to update the network weights. The experiments were performed on an Intel(R) Xeon(R) Silver 4116 CPU with an Nvidia GeForce GTX 2080TI GPU. All the models were defined and trained using the Pytorch framework.

**Table 3:** Number of facial photos in each subset in five-fold cross-validation.

**Data subset**	**WBS (*n* = 104)**	**Controls (*****n*** **=** **236)**	
		**Other syndromes (*n* = 91)**	**Healthy children (*n* = 145)**
Subset 1	21	19	29
Subset 2	21	18	29
Subset 3	21	18	29
Subset 4	21	18	29
Subset 5	20	18	29

### Face Classification Comparison of Facial Recognition Frameworks and Physicians

To compare the classification performance of these frameworks to that of physicians, two pediatricians and two pediatric cardiologists were invited to recognize WBS patients based solely on the facial photos. All facial photos (104 WBS patients and 236 control individuals) were shown to physicians and each picture was shown for 10 s without disclosing any clinical data.

### Statistical Analysis

The accuracy, precision, recall, F1 score, receiver operating characteristic (ROC) curve, and area under the ROC curve (AUC) were used to evaluate the classification performance. The accuracy, precision, recall, F1 score, and AUC of facial recognition frameworks are reported as the mean ± standard deviation (SD) (20, 21) of five testing results obtained from the cross-validation. Pearson's chi-squared-test was applied to compare the gender proportions, and an independent-sample *t*-test was used to compare the quantitative variables between the groups. All *p*-values were double-tailed, and values < 0.05 were considered statistically significant.

## Results

Five face recognition models for WBS were constructed with deep CNN architectures combined with ImageNet transfer learning. The accuracy, precision, recall, F1 score, and AUC of five models are given in Table 4. VGG-19 achieved the top value in terms of accuracy (92.7 ± 1.3%), precision (94.0 ± 5.6%), F1 score (87.2 ± 2.0%), and AUC (89.6 ± 1.3%). MobileNet-V2 had the worst classification performance with the accuracy (85.6 ± 2.9%), precision (86.4 ± 10.2%), recall (67.1 ± 19.7%), F1 score (72.6 ± 8.8%), and AUC (80.4 ± 7.1%).

**Table 4:** Accuracy, precision, recall, F1 score, and AUC of each model.

**Model**	**Accuracy %**	**Precision %**	**Recall %**	**F1 score %**	**AUC %**
VGG-16	90.9 ± 2.8	88.8 ± 8.8	**82.7** **±** **9.8**	84.7 ± 4.5	88.6 ± 3.5
VGG-19	**92.7** **±** **1.3**	**94.0** **±** **5.6**	81.7 ± 3.6	**87.2** **±** **2.0**	**89.6** **±** **1.3**
ResNet-18	87.9 ± 4.4	88.9 ± 9.2	72.2 ± 20.2	77.2 ± 12.0	83.6 ± 8.2
ResNet-34	89.1 ± 3.7	87.7 ± 12.3	78.9 ± 12.9	81.5 ± 6.8	86.3 ± 4.9
MobileNet-V2	85.6 ± 2.9	86.4 ± 10.2	67.1 ± 19.7	72.6 ± 8.8	80.4 ± 7.1

*All values are presented as the mean ± SD. Values in bold indicate the optimal performance*.

**Figure 2:**
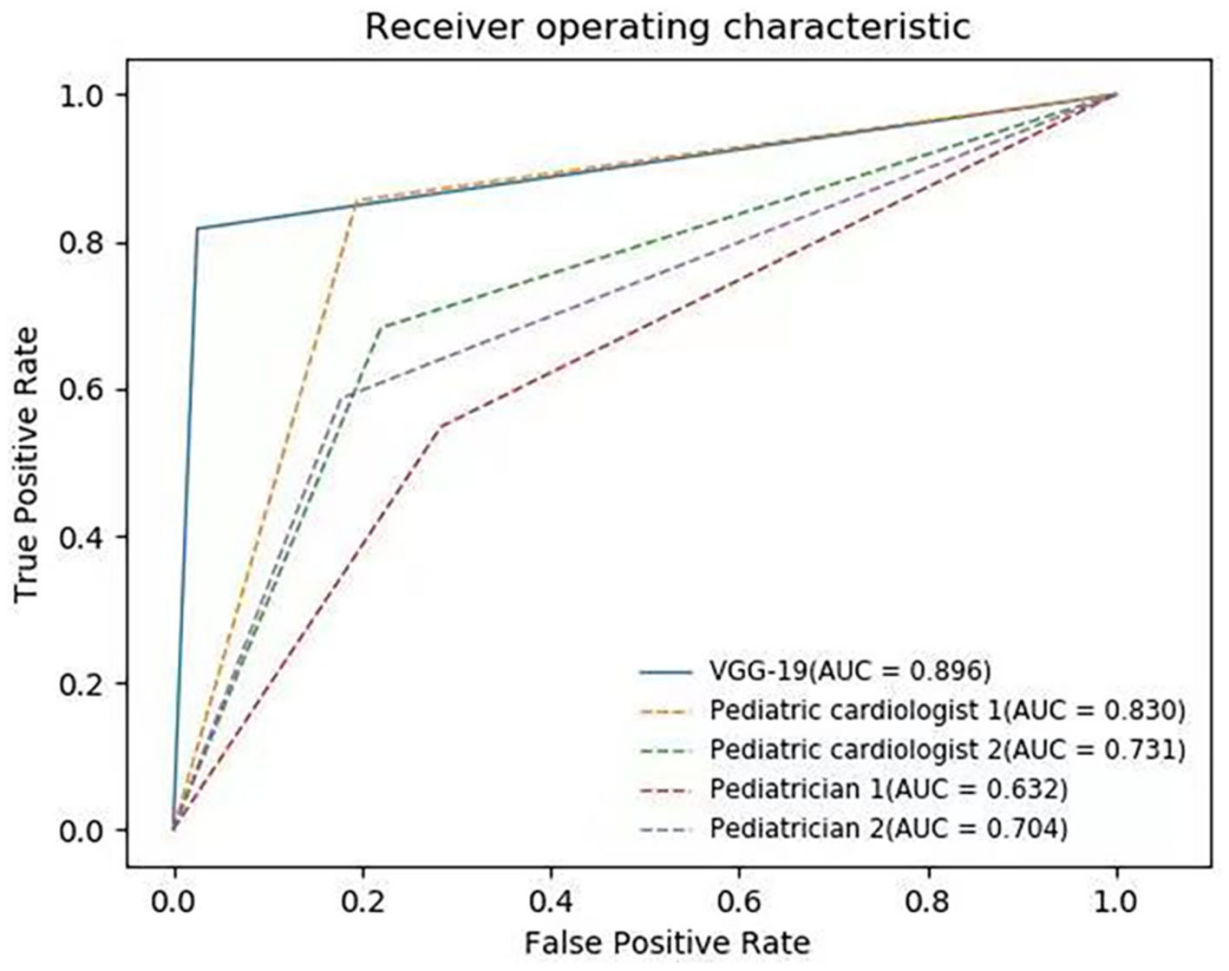
ROC curves of VGG-19 and four human experts. The average AUC of VGG-19 is 89.6%.

The classification results of four human experts are presented in Table 5. Pediatric cardiologist 1 achieved the best performance with the accuracy (82.1%), precision (65.9%), recall (85.6%), F1 score (74.5%), and AUC (83.0%). The highest accuracy of four human experts was less accurate than any of CNN-based face recognition models in our experiment. The average accuracy, precision, recall, F1 score, and AUC of the four human experts were 74.6, 57.2, 66.8, 61.5, and 72.4%, respectively. The AUCs of each human expert were inferior to the AUCs of the VGG-16 (88.6 ± 3.5%), VGG-19 (89.6 ± 1.3%), ResNet-18 (83.6 ± 8.2%), and ResNet-34 (86.3 ± 4.9%) models. The ROC curves of human experts and the VGG-19 model are shown in Figure 2.

**Table 5:** Accuracy, precision, recall, F1 score, and AUC of four human experts.

**Human experts**	**Accuracy%**	**Precision%**	**Recall%**	**F1 score %**	**AUC %**
Pediatric cardiologist 1	**82.1**	**65.9**	**85.6**	**74.5**	**83.0**
Pediatric cardiologist 2	75.0	57.7	68.3	62.6	73.1
Pediatrician 1	66.5	46.0	54.8	50.0	63.2
Pediatrician 2	75.0	59.2	58.7	58.9	70.4

*Numbers in bold indicate the best values*.

## Discussion

First described in the early 1960s, WBS is a syndrome characterized by dysmorphic facial features, supravalvular aortic stenosis, supravalvular pulmonary stenosis, peripheral pulmonary stenosis, and cognitive impairment (5, 22). WBS patients can be recognized by an experienced doctor, given the characteristic features of the patients, which include congenital heart defects and facial dysmorphism. The facial features of WBS are dysmorphic and can range from subtle to dramatic (2). Therefore, many patients are not diagnosed until the symptoms develop into severe complications. Artificial intelligence has been integrated into routine clinical practice, especially for providing diagnostic support. As one of the dominant areas of artificial intelligence, computer-aided recognition of dysmorphic faces has progressed in recent years (23).

In 2003, Loos et al. (24) first demonstrated that a computer was able to recognize a syndrome by facial resemblance, with an accuracy of 83% for WBS diagnosis. Because the graph nodes of facial pictures were all labeled by hand, the facial recognition technology reported Loos et al. was actually “semi-automatic.” In addition, the accuracy of this system has yet to be improved. With the advent of facial analysis technologies, automatic face recognition systems have rapidly developed. Kruszka et al. (25) adopted machine learning methods to automatically distinguish WBS patients from healthy individuals, achieving 92% accuracy with an Asian cohort. However, Kruszka et al. did not include patients with other genetic syndromes. As such, their system may not be capable of distinguishing WBS from other genetic syndromes in clinical practice. As the most widely used neural network, CNNs have become the top choice for automatic face recognition in recent years (7). Using deep CNNs, Gurovich et al. (10) proposed the DeepGestalt system for identifying genetic syndromes. When a facial picture is input, DeepGestalt produces a ranked list of 30 possible genetic syndromes. The DeepGestalt model was designed to identify the type of genetic syndrome a patient might have. It was not designed to determine whether or not a subject has a genetic syndrome. If an image of a healthy individual is inputted to the model, DeepGestalt still outputs a ranked list of possible genetic syndromes. In our study, five WBS face recognition frameworks based on deep CNNs were developed, and these frameworks can help pediatricians to distinguish WBS children from patients with other genetic syndromes and from healthy individuals.

The VGG-16, VGG-19, ResNet-18, ResNet-34, and MobileNet-V2 deep CNN architectures were adopted to construct different WBS facial recognition systems in the present study. These five deep CNN architectures were selected for several reasons: (a) they have the small kernels and multi-scale architectures, and can reduce the number of parameters; (b) their weights can be modified by transfer learning; and (c) they have been used widely in recent years and have achieved top performance in different challenges (26). As our results showed, the accuracy of each deep CNN model was higher than the pediatricians and pediatric cardiologists. The AUCs of each human expert were inferior to the AUCs of the VGG-16, VGG-19, ResNet-18, and ResNet-34 models. These results highlighted the practical clinical potential of automatic facial recognition based on deep CNNs for diagnosing WBS, especially in areas without access to professional clinicians or genetic testing. In our study, VGG-19 model achieved the best performance, followed by the VGG-16 model. VGGNet is characterized by its simplicity in using only 3 × 3 convolutional layers stacked on top of each other in increasing depth. The increased depth and smaller kernel can promote the fitting capacity and improve the classification accuracy. The facial recognition framework based on VGGNet could provide precise information for clinicians in WBS diagnosis.

WBS is a rare disease with an incidence rate of about 0.01%. Due to the insufficiency of WBS facial picture data, it is challenging to effectively train a deep CNN model for WBS facial recognition that avoids the over-fitting problem. This issue can be bypassed using transfer learning, by adjusting a well-trained network on a certain domain to another related one (27). The ImageNet dataset has become the main choice for transfer learning among artificial intelligence practitioners in medical tasks. In this study, CNN architectures obtained the initial weights through ImageNet transfer learning. The ImageNet dataset has 13 million natural images of 1,000 different classes (28). The recognition models in the present study learn comprehensive natural features from the ImageNet dataset, followed by knowledge transfer to WBS facial classification through fine-tuning. Therefore, our WBS facial recognition models achieved good classification performance on a relatively small dataset of only 340 pictures. Transfer learning technology can be generalized to other rare genetic syndromes with facial dysmorphism. It may be feasible to leverage CNNs to learn the ImageNet dataset with corresponding weight transfers for other genetic syndromes using a small-scale dataset.

Although we achieved considerable performance accuracy and efficiency, there were some limitations to our study. First, the age of the participants ranged from 1 month to 14 years. Therefore, this recognition system might not be useful for WBS cases outside of this age group. Second, in the control group, 13 healthy children underwent next-generation sequencing or array comparative genomic hybridization to exclude WBS, and 132 healthy children were consulted by two clinical geneticists to exclude genetic syndromes without any confirmation through genetic testing. Thus, some of the controls might have had undiagnosed WBS. However, this probability is extremely small, and none of them showed any manifestation of WBS.

In conclusion, this study highlighted the possibility of using deep CNNs for WBS facial identification in clinical practice. The facial recognition framework based on VGG-19 could play a prominent role in WBS diagnosis. Transfer learning technology can help construct facial recognition models of genetic syndromes with small-scale datasets.

## Data Availability Statement

The original contributions presented in the study are included in the article/Supplementary Material, further inquiries can be directed to the corresponding authors.

## Ethics Statement

The studies involving human participants were reviewed and approved by Research Ethics Committee of Guangdong Provincial People's Hospital, Guangdong Academy of Medical Sciences (Project No. KY2020-033-01). Written informed consent to participate in this study was provided by the participants' legal guardian/next of kin. Written informed consent was obtained from the individual(s), and minor(s)' legal guardian/next of kin, for the publication of any potentially identifiable images or data included in this article.

## Author Contributions

HL and S-SW contributed to manuscript writing. S-SW, JZ, and Z-WZ contributed to conception and design. HL, Z-HM, HY, Z-FZ, DH, LW, M-YL, Y-YZ, and X-WX collected and organized the database. All authors contributed to manuscript revision, read, and approved the submitted version.

## Conflict of Interest

The authors declare that the research was conducted in the absence of any commercial or financial relationships that could be construed as a potential conflict of interest.
