# Supplementary material for: Automatic Facial Recognition of Williams-Beuren Syndrome Based on Deep Convolutional Neural Networks
Source: Front Pediatr. 2021 May 19;9:648255. doi: 10.3389/fped.2021.648255 (PMC8170407; doi:10.3389/fped.2021.648255)
Supplement: Supplementary file 1 [file Data_Sheet_1.PDF]

Supplementary Material

1 Supplementary Figures

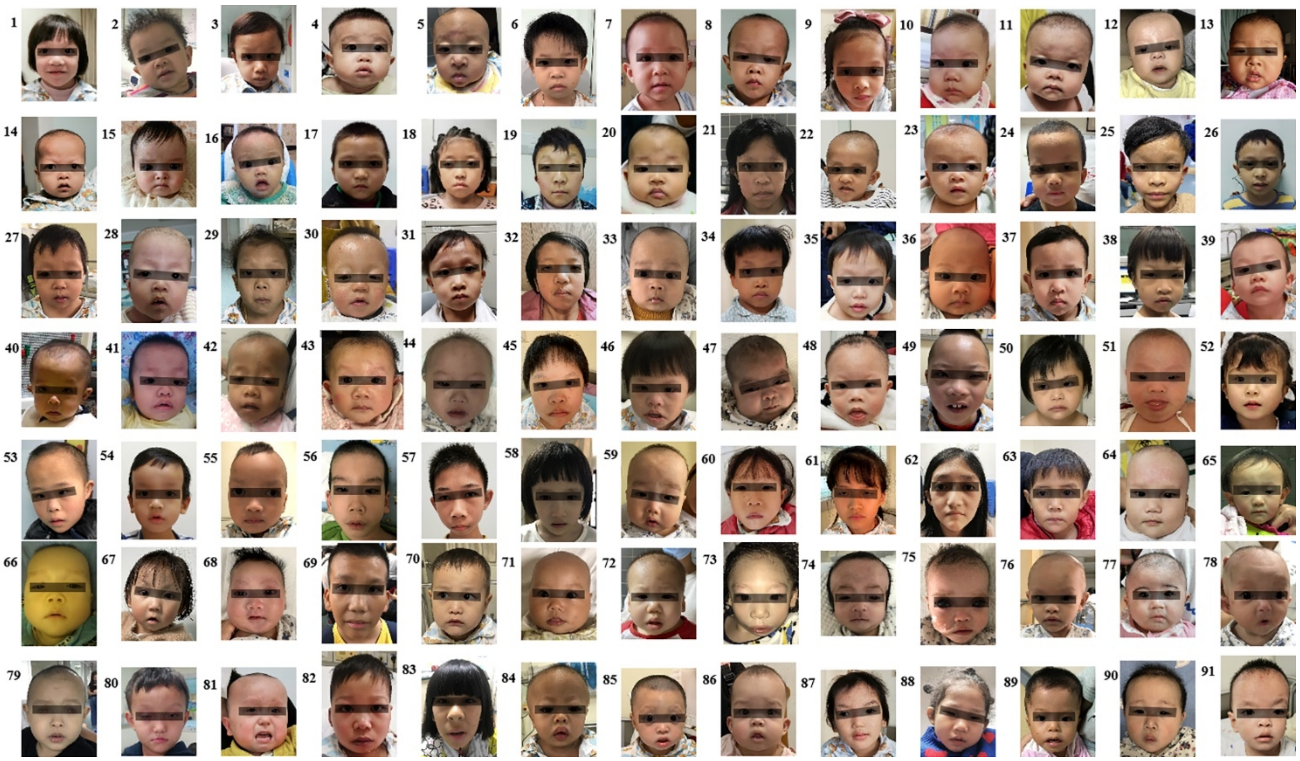

Supplementary Figure 1. Facial appearance of other genetic syndromes (91 cases): Noonan syndrome (1-43), Down syndrome (44-53), Loeys–Dietz syndrome (54-57), DiGeorge syndrome (58-60), Marfan's syndrome (61-63), Alagille syndrome (64-66), and other rare syndromes (67-91). The black bar is used to protect privacy.
